# Moving beyond anti-amyloid therapy for the prevention and treatment of Alzheimer’s disease

**DOI:** 10.1186/s12883-014-0169-0

**Published:** 2014-09-02

**Authors:** Michaela A Castello, John David Jeppson, Salvador Soriano

**Affiliations:** Department of Anatomy, Loma Linda University School of Medicine, Evans Hall B08, 24785 Stewart Street, Loma Linda, 92354 CA USA

**Keywords:** Late onset Alzheimer’s disease, Amyloid cascade hypothesis, Anti-amyloid therapy, Amyloid beta, Familial Alzheimer’s disease, Solanezumab, Bapineuzumab, Cholesterol metabolism, Dementia, Neurodegeneration

## Abstract

**Background:**

High-profile Phase 3 clinical trials of bapineuzumab and solanezumab, antibodies targeted at amyloid-beta (Aβ) removal, have failed to meet their primary endpoints. Neither drug improves clinical outcomes in patients with late onset AD, joining a long list of unsuccessful attempts to treat AD with anti-amyloid therapies.

**Discussion:**

These therapies are based on the assumption that Aβ accumulation is the primary pathogenic trigger of AD. Current evidence suggests that Aβ may actually accumulate as part of an adaptive response to long-term chronic brain stress stimuli that would make more suitable candidates for therapeutic intervention.

**Summary:**

At this juncture it is no longer unreasonable to suggest that further iterations of anti-Aβ therapies should be halted. Clinicians and researchers should instead direct their attention toward greater understanding of the biological function of Aβ both in healthy and demented brains, as well as the involvement of long-term chronic exposure to stress in the etiology of AD.

**Electronic supplementary material:**

The online version of this article (doi:10.1186/s12883-014-0169-0) contains supplementary material, which is available to authorized users.

## Background

According to reports published in the *New England Journal of Medicine*, the phase 3 clinical trials of two high-profile Alzheimer’s disease (AD) antibodies against the aggregation-prone peptide amyloid beta (Aβ), bapineuzumab and solanezumab, have failed to improve clinical outcomes in patients with late onset AD [1]-[3]. Along with their predecessors, these treatments have been informed by the long-standing amyloid hypothesis, and are the latest examples in a long list of unsuccessful attempts to treat AD with anti-amyloid therapies. Along with a chorus of others, we have previously argued against the assumption that Aβ accumulation is the primary early pathogenic trigger of AD [4]-[8]. An unintended consequence of that assumption, which contributes to the continued failure of anti-amyloid clinical trials, is that affirmative diagnosis of AD-type dementia can only occur when the presence of Aβ accumulation in the brain is confirmed. However, recent imaging studies confirm previous observations of Aβ accumulation in a significant proportion of non-demented individuals [4],[9],[10]. Conversely, a sizable proportion of patients *clinically diagnosed with AD* do not display Aβ accumulation-even though neurodegeneration is in progress [4],[11]. Remarkably, rather than concluding that Aβ status is not a reliable marker for the early stages of clinical AD, a consensus has been reached in which clinically diagnosed AD patients without Aβ are classified as not suffering from AD. This line of thought is not scientifically warranted, as there is no evidence to assume that clinical AD cases with and without Aβ accumulation are etiologically different. Nevertheless, it has been used, in the EXPEDITION 3 phase of the ongoing solanezumab trial, to justify the exclusion of approximately 25% of patients in the study-all of whom were clinically diagnosed with mild AD, but whose imaging data showed no Aβ accumulation [1],[3].

## Discussion

We submit that such course of action is logically flawed on two different fronts. Firstly, current imaging methods cannot detect the soluble Aβ oligomers that solanezumab is thought to remove but that are, according to the amyloid hypothesis itself, the *bona fide* pathogenic trigger of the disease [12]-[14]. Thus, by eliminating all patients diagnosed with clinical AD but lacking Aβ plaques, all the subjects that would potentially benefit from the trial are effectively removed. Secondly, there is no obvious rationale for following patients in whom Aβ plaques are already detectable, since the presence of those plaques occurs, according to the amyloid hypothesis itself, too late in the disease for treatment to be effective and does not necessarily correlate with neurodegeneration [3],[12]-[18]. In effect, the current course of action helps to perpetuate a tautological argument: the a priori assumption that Aβ is the cause of AD is used to reject any clinical case in which no Aβ increase is apparent.

**Figure 1:**
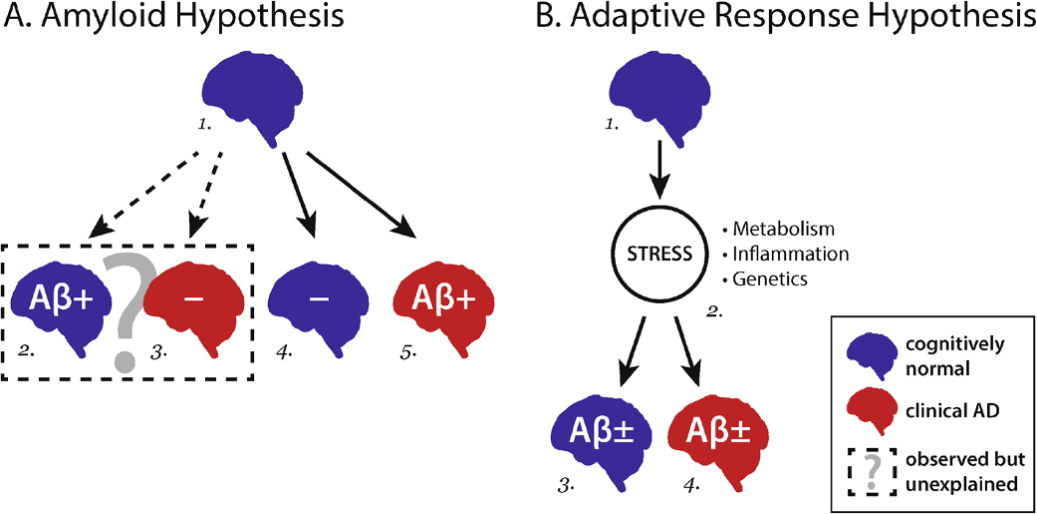
**Comparison of the amyloid and adaptive response hypotheses.** **A**. Amyloid Hypothesis Cognitive tests and amyloid imaging separate the total population into four distinct groups (1). These groups are: Normal Cognition (NC; ***4***), NC with Aβ accumulation (NC-Aβ; ***2***), Neurodegeneration-First AD (NDF-AD; ***3***), and Amyloid-First AD (AF-AD; ***5***). Under this hypothesis, only the AF-AD and NC groups ***(4,5)*** are going to be studied moving forward in EXPEDITION 3 as disease state and control, whereas the NC-Aβ and NDF-AD groups ***(2,3)*** are ignored, as they cannot be explained and do not fit the paradigm. **B**. *Adaptive Response Hypothesis* The total population ***(1)*** is differentiated by a set of stress variables ***(2)*** which may include, but are not limited to, oxidative stress, metabolism dysregulation (cholesterol homeostasis, insulin resistance, etc.), genetic factors, and inflammation response. These variables elicit an adaptive response in the brain and, depending on the nature and intensity of such response, the population falls into two groups, either Normal Cognition (NC) ***(3)*** or AD ***(4)***, both of which contain Aβ positive and negative subpopulations.

Figure 1 further illustrates what we believe is the flawed rationale on which anti-amyloid clinical trials are based. Cognitive status assessment and Aβ imaging data lead, according to the amyloid hypothesis, to a division of the population into four distinct groups (Figure 1A), which are: patients who are cognitively healthy (normal cognition, NC; Figure 1A, 4), patients who are cognitively healthy but accumulate Aβ (normal cognition with Aβ, NC-Aβ; Figure 1A, 2), patients with neurodegeneration who have clinical AD symptoms but no Aβ accumulation (neurodegeneration-first AD, NDF-AD; Figure 1A, 3), and finally, patients who have neurodegeneration, clinical AD symptoms, and Aβ accumulation (Aβ-first AD, AF-AD; Figure 1A, 5) [3],[4],[11]. According to the amyloid hypothesis, of all the observed populations, only the latter can be considered, by definition, as suffering from dementia of the AD type, and only group 4 should be considered as appropriate normal cognition controls in clinical trials.

The ongoing insistence on failing anti-amyloid therapies is anchored on the belief that late onset AD primarily develops from aberrant Aβ biology that results in its accumulation. When the amyloid hypothesis was formed, strong evidence clearly supported that assumption: Not only do all familial cases of AD involve APP mutations that cause dysregulated Aβ production, cases of trisomy 21 (Down syndrome; DS) in which APP was overexpressed also exhibit Aβ plaque formation identical to that of AD patients [19]-[21]. Since both FAD and DS exhibit pathology clearly linked to Aβ production, late onset AD-which also has abnormal Aβ-must also begin with Aβ. Such a conclusion has subsequently been supported by innumerable animal and cell culture studies in which pathology is induced by Aβ and rescued by its removal [22]-[25]. Given these findings, the thinking currently guiding AD clinical trials concludes that Aβ-modifying therapies simply *must* be capable of preventing late-onset AD if administered correctly.

However, this line of reasoning does not account for numerous other current observations. For example, while all cases of FAD can be linked to a relatively small number of mutations directly affecting APP processing, this is never the case with late-onset AD [26],[27]. In fact, in the largest genetic analyses of late-onset AD to date, the polymorphisms commonly observed are nearly all associated with cholesterol metabolism, endocytosis (an essential part of cholesterol processing), and inflammation [28]-[30] This evidence, together with imaging studies showing that Aβ accumulation can be uncoupled from disease initiation, strongly argue against Aβ as an early pathogenic trigger of late onset AD and, therefore, as a suitable therapeutic target [9]-[11].

In our view, resolving the apparent contradictions in evidence begins with abandoning the assumption that FAD and late onset AD are etiologically comparable. Doing so will help create the right context for the study of the role of Aβ in health and disease, a role we do not currently understand. In that regard, note that virtually every experimental in vitro and in vivo model demonstrating Aβ harm and subsequent improvement upon its removal is, at best, a model of FAD. By definition, these models begin with the overexpression of Aβ itself, a pathogenic course that does not occur in late onset AD [22]-[25]. In contrast, if FAD were considered not as an accelerated version of late onset AD, but rather as a subset of AD presentations that is etiologically different, we could begin to explore the *bona fide* pathogenic triggers of late onset AD and design evidence-based therapies.

In that regard, several hypotheses that are not amyloid-centric have been proposed, although few have gained significant traction [6],[31]-[35]. Unlike in the past, however, numerous independent researchers have now gathered sufficient information to strongly support a reworked conceptualization of late onset AD. Our recently proposed Adaptive Response Hypothesis synthesizes this work, proposing that Aβ may accumulate as part of an adaptive response to chronic brain stress stimuli [6]. These stress stimuli constitute the *bona fide* pathogenic triggers of late onset AD and, therefore, would be suitable candidates for therapeutic intervention [5]-[7],[32],[36]. In this model, illustrated in Figure 1B, the total population (Figure 1B, 1) can be affected by chronic stress stimuli (Figure 1B, 2) which may include, but are not limited to, oxidative stress, metabolic dysregulation (cholesterol homeostasis, insulin resistance, etc.), genetic factors, and inflammatory response [7],[36]. Each of these stimuli is capable of eliciting a response in which Aβ is produced, and the nature of that response (not the total amount of Aβ that may accumulate in parallel) determines progression into clinical AD [5],[6]. Ultimately this leads to the observed division, shown in Figure 1B, into individuals with normal cognition (NC; Figure 1B, 3) and those clinically diagnosed with AD (AD; Figure 1B, 4), both of which may be further divided into Aβ positive and Aβ negative subpopulations [6],[32].

According to this view, therapeutic approaches must address the biology of the chronic stressors that initiate the disease, not the Aβ accumulation that (unlike in FAD) may, or may not, occur during the course of the disease. This offers numerous potential avenues to explore in the battle against AD. In fact, research into aging, cholesterol regulation, and metabolic disorders such as diabetes all can potentially be applied to AD. Conceiving of the disease in this open-ended, systemic fashion will allow clinicians and scientists to identify new patterns and possibilities for therapy. For example, early research has shown that metabolism in the AD brain is aberrant in ways that are not currently looked for in the periphery [37],[38]. Following this pathway, early treatment intranasal insulin has actually shown some promise in treating cognitive decline [38]. Similarly, might drugs enhancing neural plasticity empower the brain’s stress response in old age? [39].

Finally, it is worth noting that anti-amyloid therapies may not simply result in neutral outcomes. Our hypothesis predicts that Aβ removal will interfere with brain homeostasis, and mounting evidence suggests that well-regulated Aβ is important for healthy brain functions such as memory formation-a function that is critical to clinical outcome measurements [40]-[43]. At the same time, even the most recent bapineuzumab trial continues to be limited by edema formation, a symptom highly associated with cerebral amyloid angiopathy, the damaging vascular amyloid deposition that often co-occurs with AD [2],[3],[44]. Thus, the possibility must be considered that current therapies designed around the bulk removal of Aβ may not simply fail, but be actively harmful by hindering the very functionality they hope to preserve.

In summary, millions of research dollars, both private and public, are annually expended on anti-Aβ therapies that do not work and are based on a logically flawed hypothesis. At this point in time it is no longer unreasonable to suggest that further iterations of anti-Aβ therapies may not be in the best interest of late onset AD patients. Clinicians and researchers should instead direct their attention toward better understanding of the biological function of Aβ in the healthy brain, and the ways in which chronic stress over decades can negatively affect the brain.

## Summary

- The authors contend that the amyloid cascade hypothesis is no longer supported by the majority of experimental evidence
- Proposed elimination of patients from EXPEDITION 3 of the solanezumab Phase III trial based on Aβ imaging is fundamentally flawed
- Aβ-centric therapeutic studies promote a tautological definition of Alzheimer’s disease in which the a priori assumption that Aβ is the primary causative factor is used to exclude patients exhibiting contrary symptoms
- An adaptive response hypothesis summarizes a diverse body of experimental evidence and is able to account for all AD-related presentations
- Such a hypothesis provides new opportunities for research and potential therapies that the amyloid cascade hypothesis does not

## Authors’ original submitted files for images

Below are the links to the authors’ original submitted files for images. Authors’ original file for figure 1

## Abbreviations

AD: Alzheimer’s disease
FAD: Familial AD
Aβ: Amyloid beta
NC: Normal cognition
NC-Aβ: Normal cognition with Aβ
NDF-AD: Neurodegeneration-first AD
AF-AD: Aβ-first AD
